# Trends in prevalence of hip osteoarthritis over a 10-year period in Japan: The ROAD study 2005–2015

**DOI:** 10.1016/j.ocarto.2022.100285

**Published:** 2022-06-22

**Authors:** Toshiko Iidaka, Chiaki Horii, Shigeyuki Muraki, Hiroyuki Oka, Hiroshi Kawaguchi, Kozo Nakamura, Toru Akune, Sakae Tanaka, Noriko Yoshimura

**Affiliations:** a Department of Preventive Medicine for Locomotive Organ Disorders, 22nd Century Medical & Research Center, Faculty of Medicine, University of Tokyo, Tokyo, Japan; b Department of Orthopaedic Surgery, Faculty of Medicine, University of Tokyo, Tokyo, Japan; c Department of Medical Research and Management for Musculoskeletal Pain, 22nd Century Medical & Research Center, Faculty of Medicine, University of Tokyo, Tokyo, Japan; d Department of Orthopaedic Surgery, Tokyo Neurological Center, Tokyo, Japan; e Towa Hospital, Tokyo, Japan; f National Rehabilitation Center for Persons with Disabilities, Saitama, Japan

**Keywords:** Osteoarthritis, Hip, Prevalence, Cohort study

## Abstract

**Objective:**

The trends in prevalence of hip osteoarthritis (OA) over a 10-year period among Japanese men and women were investigated using the data from the Research on Osteoarthritis/osteoporosis Against Disability study.

**Design:**

We analyzed the data of 2924 baseline survey participants (1026 men, 1898 women) aged 40–89 years (mean 70.7 years) residing in urban, mountainous, and coastal communities who were surveyed in 2005–2007. We compared these data with that of 2347 participants (726 men, 1621 women) aged 40–89 years (mean 69.2 years) from the fourth survey in 2015–2016. The fourth survey invited participants to attend follow-ups for baseline survey and recruited new participants. After scoring the radiographs using the Kellgren/Lawrence (K/L) grading system, hip OA was defined as a K/L score ≥2.

**Results:**

The prevalence of radiographic hip OA was 18.4% and 14.4% in the baseline survey, and 16.0% and 10.7% in the fourth survey among men and women, respectively. Among the participants aged 40–69 years, the prevalence of radiographic hip OA was significantly lower during the fourth survey than during the baseline survey. Whereas, among elderly men aged 70–79 years, radiographic hip OA was significantly less prevalent during the baseline survey than during the fourth survey. From the logistic regression analysis results, radiographic hip OA was significantly less prevalent during the fourth survey than during the baseline survey (odds ratio: 0.55, 95% confidence interval: 0.46–0.65).

**Conclusion:**

At a 10-year interval, the prevalence of radiographic hip OA shows an improving trend.

## 1 Introduction

Hip osteoarthritis (OA) leads to chronic disability in the elderly and, therefore, is regarded as a major global public health problem [1,2]. With the aging of the Japanese society, the number of individuals requiring support or long-term care has increased. The recent National Livelihood Survey by the Ministry of Health, Labour and Welfare in Japan [3] determined dementia (17.6%), cardiovascular disease (16.1%), frailty (12.8%), falls/osteoporotic fractures (12.5%), and OA (10.8%) as the leading causes of disability. Therefore, strategies to prevent and treat hip OA are urgently necessary. Generally, to prevent a disease, it is necessary to clarify epidemiological data, such as the number of patients (prevalence), how many patients will be incident per year (incidence rate), which are the risk factors that are associated with a disease, and the natural course of the disease. It is difficult to design rational clinical and public health approaches for the diagnosis, evaluation, and prevention of disease without such epidemiological data. However, musculoskeletal diseases, such as hip OA progress slowly; moreover, as the disease course is long, not all patients who have musculoskeletal diseases are observed in a clinic. Therefore, surveys at medical facilities have limitations for early detection of diseases. Prospective longitudinal population-based surveys are needed to estimate the epidemiological data for preventing musculoskeletal diseases. The prevalence and incidence rate of radiographic hip OA has not been well reported [[4], [5], [6], [7], [8], [9], [10], [11]]; even the long-term prevalence of radiographic hip OA remains to be evaluated, whereas the analysis of the long-term prevalence of radiographic hip OA would provide important data that could be used for developing hip OA prevention strategies.

To establish epidemiologic indices for evaluating bone and joint diseases, the Research on Osteoarthritis/Osteoporosis Against Disability (ROAD) study, a large-scale nationwide cohort study, was performed in 2005. We had previously reported the prevalence of radiographic hip OA by using the baseline survey data, based on the Kellgren/Lawrence (K/L) grading system [12]. Thereafter, we clarified the incidence and progression rate of radiographic hip OA along with their risk factors using data of the 10-year longitudinal survey on Japanese men and women [13].

We investigated the epidemiological data of radiographic hip OA, such as prevalence, incidence rate, and risk factors. In Japan, the proportion of the ageing population is increasing [14]; in contrast, Suzuki, et al. compared physical constitution and physical function in the elderly in 2007 and 2017 and found that the phenomenon of rejuvenation is occurring among Japanese older adults [15]. Therefore, we hypothesized that the prevalence of radiographic hip OA might have reduced due to rejuvenation of physical constitution and physical function. Interpreting long-term changes in radiographic hip OA is an epidemiological data gathering tool. These data can help develop strategies to prevent hip OA. In the ROAD study, the additional follow-up survey had approximately 3000 participants, including follow-up and new participants who were recruited as volunteers. The quality research data of the ROAD study have been maintained as one cross-sectional and one longitudinal survey. Using the data of both these large-scale cross-sectional cohorts (the baseline and 10-year survey), we investigated the long-term trend of hip OA. We also compared the prevalence of radiographic hip OA in the same age group in 10-year increments without participant overlapping between both surveys as a birth cohort [[16], [17], [18]].

Thus, this study examined the trends in prevalence of hip OA over a 10-year period among Japanese men and women by retrieving data from the large population cohort of the ROAD study.

## 2 Method

The present study was retrieved from the ROAD cohort, which was established in 2005 and is a national prospective study of OA consisting of population-based cohorts from several communities in Japan (cohorts described elsewhere) [12,[19], [20], [21], [22]]. In brief, between 2005 and 2007, a baseline database that included clinical and genetic information of 3040 individuals (1061 men and 1979 women; mean age, 70.3 years; range, 23–95 years) was created. The participants were recruited from resident registration listings in three communities with different characteristics: 1350 participants from an urban region in Itabashi, Tokyo; 864 participants from a mountainous region in Hidakagawa, Wakayama; and 826 participants from a coastal region in Taiji, Wakayama. Participants who could walk to the survey site, report data, and understand and sign the informed consent form were included. All participants provided written informed consent, and the ethics committees of the University of Tokyo and the Tokyo Metropolitan Geriatric Medical Center approved the study.

**Fig. 1:**
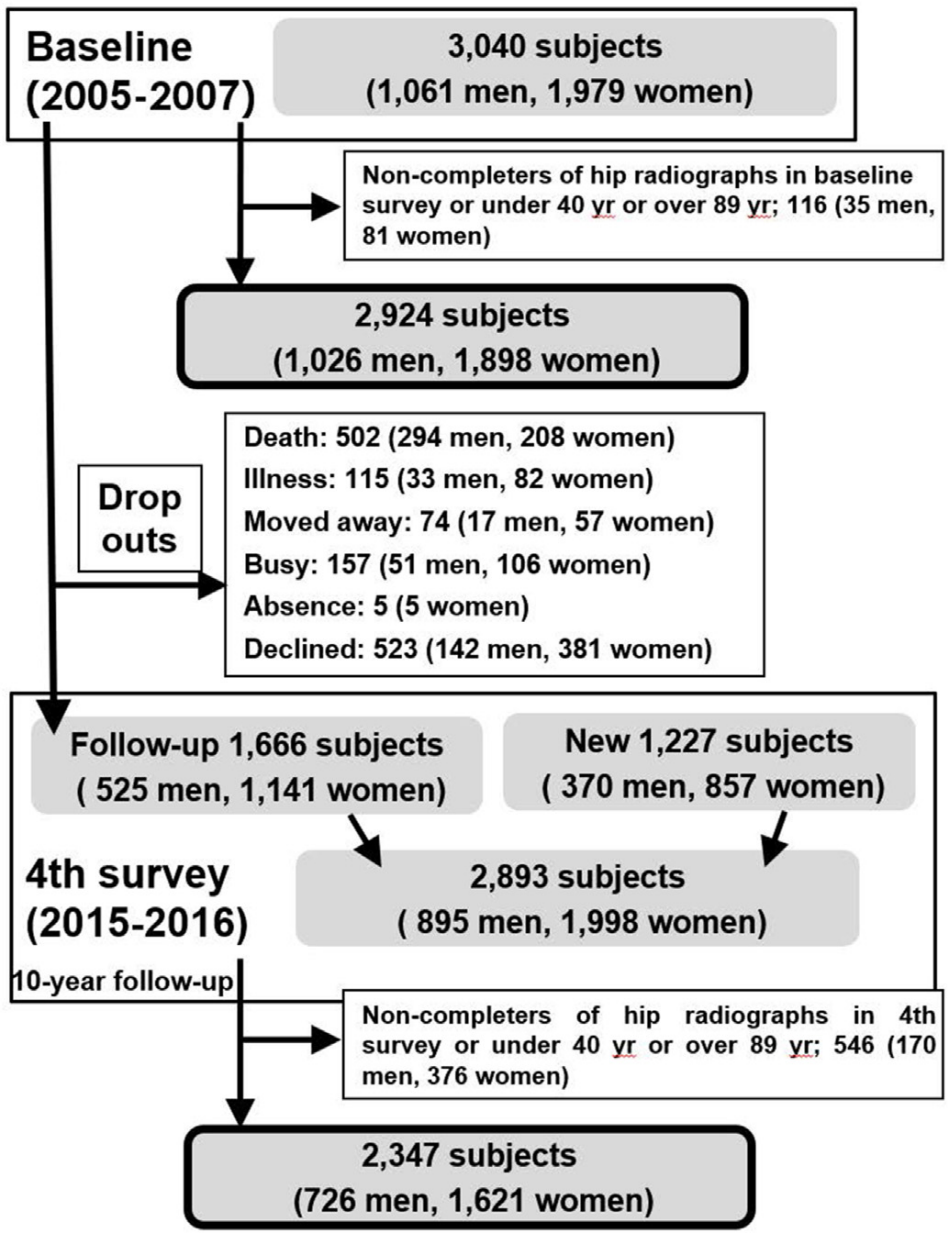
Flowchart of participant selection.

After the baseline survey, the same communities were resurveyed during 2008–2010; thereafter, the third and fourth surveys were conducted during 2012–2013 and 2015–2016 [13], wherein the baseline examinations were repeated. The follow-up rates were 81.7%, 66.3%, and 54.8% in the second, third, and fourth surveys, respectively. Each follow-up survey invited participants to attend follow-ups for these surveys and also recruited new participants from each region. Especially, the fourth survey of the ROAD study was a 10-year follow-up survey; therefore, we recruited many new participants as volunteers to set up a new baseline survey by using publications in each region. A total of 1666 individuals (525 men and 1141 women) participated in both the baseline and fourth surveys. A total of 1227 individuals (370 men and 857 women) were the new participants in the fourth survey. Thus, a total of 2893 individuals (895 men and 1998 women; mean age, 68.9 years; range, 18–97 years) participated in the fourth survey. For this study, we selected participants aged 40–89 years from the baseline and the fourth surveys (Fig. 1).

### 2.1 Examination performed during baseline and fourth survey

1) Questionnaire

The participants completed an interviewer-administered questionnaire consisting of questions on lifestyle, occupation, smoking habits, alcohol consumption, family history, medical history, physical activity, reproductive history, and health-related quality-of-life.

2) Anthropometric measurements

All participants underwent height and weight measurements that were used for calculating the body mass index (BMI) as weight (kg)/height^2^ (m^2^). Handgrip strengths of both arms were measured using a handgrip dynamometer (Toei Light Co., Ltd., Saitama, Japan). The larger value was noted as the maximum handgrip strength.

3) Radiographic assessment

All participants underwent plain radiography in the anteroposterior pelvic view with a weight-bearing position having the feet internally rotated. Fluoroscopic guidance with a horizontal anteroposterior X-ray beam was used to properly visualize the joint space. The tube-to-film distance was 110 ​cm in all cases. All hip radiographs were read by a well-experienced orthopedist (TI) blinded to the clinical status of participants, and K/L grade was defined using the K/L atlas for overall hip radiographic grades [23]. In the K/L grading system, radiographs are scored from 0 to 4 points, with higher grades being associated with more severe OA. Good intra- and inter-variabilities have been documented [12].

Radiographic hip OA was defined as a K/L grade ≥2 (at least probable joint space narrowing in the superolateral or superomedial hip joint with an osteophyte present). We defined an individual as having radiographic hip OA if at least one hip joint was affected. Individuals who underwent total hip arthroplasty were defined as having K/L grade 4 OA in that joint (baseline survey: n ​= ​13 individuals, 18 hips; fourth survey: n ​= ​10 individuals, 15 hips).

### 2.2 Statistical analyses

The trends in age, height, weight, and BMI between the baseline survey (2005–2007) and the fourth survey (2015–2016) were examined using t-tests, similar to that in each age group. The χ^2^ test was used for comparing trends in proportions, and current smoking and drinking habits between the baseline and the fourth surveys. To test the association between the presence of radiographic hip OA in the baseline and the fourth survey with potential associated factors, multivariable logistic regression analysis was used. In the analysis, the presence of radiographic hip OA was used as the objective variable (0, group in 2005–2007; 1, group in 2015–2016), and explanatory variables were selected in addition to the basic characteristics (i.e., age, sex, BMI, and residence area). Data analyses were performed using JMP version 11.0 (SAS Institute, Cary, NC, USA).

## 3 Results

Of 3040 participants in the baseline survey (2005–2007), 62 (2.0%) did not undergo plain radiography, one (0.03%) had acute bilateral hip fractures, two (0.07%) could not read, 45 (1.48%) were aged <40 years, and six (0.20%) were aged >90 years; hence, they were excluded (Fig. 1). The remaining 2924 individuals (96.2%; 1026 men and 1898 women) aged 40–89 years (mean 70.7 years) were included in this study. Of 2893 participants in the fourth survey (2015–2016), nine (0.31%) did not undergo plain radiography, two (0.07%) had acute bilateral hip fractures, 60 (2.07%) were aged <40 years, 58 (2.0%) were aged >90 years, and 417 (14.4%) submitted only questionnaires; these individuals were excluded. The remaining 2347 individuals (81.1%; 726 men and 1621 women) aged 40–89 years (mean age, 69.2) were included in this study (Table 1). The participants in the fourth survey were significantly taller than those in the baseline survey (*p* ​< ​0.0001), and handgrip strength in the fourth survey was significantly stronger than that in the baseline survey (*p* ​< ​0.0001).

**Table 1:** Baseline and fourth survey characteristics of the participants.

	Baseline survey (2005–2007)			Fourth survey (2015–2016)		
	Overall	Men	Women	Overall	Men	Women
Number of participants	2924	1026	1898	2347	726	1621
Age (years)	70.7 ​± ​10.2	71.4 ​± ​9.9	70.3 ​± ​10.4	69.2 ​± ​12.3‡	69.0 ​± ​12.6‡	69.2 ​± ​12.1†
Height (cm)	154.1 ​± ​8.9	162.4 ​± ​6.6	149.6 ​± ​6.4	155.8 ​± ​9.4‡	165.5 ​± ​6.9‡	151.4 ​± ​6.8‡
Weight (kg)	54.9 ​± ​10.3	61.3 ​± ​9.9	51.4 ​± ​8.7	55.5 ​± ​11.3∗	64.1 ​± ​11.1‡	51.7 ​± ​9.0
BMI (kg/m2)	23.0 ​± ​3.3	23.2 ​± ​3.0	22.9 ​± ​3.5	22.8 ​± ​3.5†	23.3 ​± ​3.3	22.5 ​± ​3.5†
Current smoking habit (%)	11.6	25.5	3.5	7.7§§	19.2§	2.6
Current alcohol drinking habit (%)	40	64.7	25.6	39.7	66.8	27.5
Grip strength (maximum) (kg)	25.3 ​± ​9.0	33.0 ​± ​8.6	21.2 ​± ​6.1	28.9 ​± ​9.9‡	39.8 ​± ​9.0‡	24.0 ​± ​5.4‡

Data are means ±SD.

BMI, body mass index.

^∗^p < 0.05 vs the baseline survey in the corresponding group by non-paired t-test.

†p < 0.001 vs the baseline survey in the corresponding group by non-paired t-test.

‡p < 0.0001 vs the baseline survey in the corresponding group by non-paired t-test.

§p < 0.001 vs the baseline survey residents in the corresponding group by χ^2^ test.

§§p < 0.0001 vs the baseline survey residents in the corresponding group by χ^2^ test.

### 3.1 Trends in prevalence of hip OA over a 10-year period

**Fig. 2:**
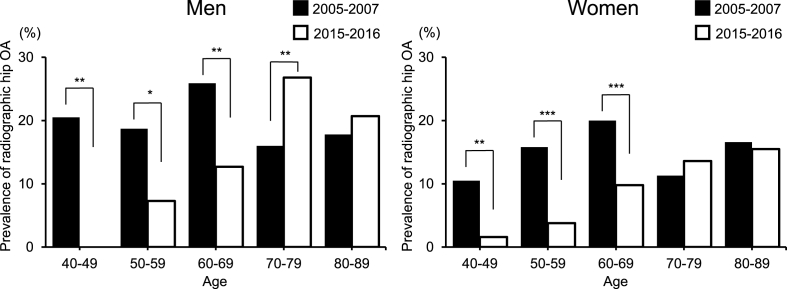
Prevalence (percentage) of radiographic hip osteoarthritis in each age strata (40–49, 50–59, 60–69, 70–79, and 80–89 years) at 2005–2007 and 2015–2016. ∗*p* ​< ​0.05, ∗∗*p* ​< ​0.01, ∗∗∗*p* ​< ​0.0001.

The prevalence of radiographic hip OA during the baseline and the fourth survey period was 15.8% and 12.4% in the whole cohort, 18.4% and 16.0% in men, and 14.4% and 10.7% in women, respectively. Fig. 2 shows the age-sex distribution of the prevalence of radiographic hip OA during the baseline and the fourth survey periods. During the baseline survey, the prevalence of radiographic hip OA in men aged 40–49, 50–59, 60–69, 70–79, and 80–89 years were 20.5%, 18.7%, 25.9%, 16.0%, and 17.8%, respectively. The corresponding rates in women were 10.5%, 15.8%, 20.0%, 11.3%, and 16.6%, respectively. During the fourth survey, the prevalence of radiographic hip OA were 0%, 7.3%, 12.7%, 26.8%, 20.7%, and 17.6% in men, and 1.6%, 3.8%, 9.8%, 13.6%, 15.5%, and 12.5% in women, respectively. Across both sexes, radiographic hip OA was less prevalent during the fourth survey in individuals aged 40–49 (men: *p* ​= ​0.0003, women: *p* ​= ​0.0041), 50–59 (men: *p* ​= ​0.0130, women: *p* ​< ​0.0001), and 60–69 years (men: *p* ​= ​0.0011, women: *p* ​< ​0.0001) than that during the baseline survey. Especially, in men aged 70–79 years, the prevalence of radiographic hip OA was significantly higher during the fourth survey period than that during the baseline survey (*p* ​= ​0.0024).

Table 2 shows the results of the logistic regression analysis after adjusting for age, sex, BMI, and communities for comparing the prevalence of radiographic hip OA during the baseline and the fourth survey periods. Radiographic hip OA was significantly less prevalent during the period of the fourth survey than during the baseline survey.

**Table 2:** Comparison for the prevalence of the radiographic hip OA between baseline and fourth survey.

	Hip OA (−)	Hip OA (+)	*p-value*	Crude OR (95%Cl)		Adjust OR^a^ (95%Cl)	
Baseline survey (%)	84.2	15.8	0.0004	Reference		Reference	
(2005–2007)							
Fourth survey (%)	87.6	12.4		0.75	(0.64–0.88)	0.55	(0.46–0.65)
(2015–2016)							

a Adjusted odds ratios (ORs) were calculated by logistic regression analysis after adjustment for age, sex, BMI and communities. OA, osteoarthritis; 95%CI, 95% confidence interval; BMI, body mass index.

## 4 Discussion

To our knowledge, this is the first large-scale population-based study that examines the trends in prevalence of hip OA over a 10-year period in the Japanese population. We reported a significantly lower prevalence of the condition in younger participants, including those aged ≤60 years, than that reported 10 years prior.

In this study, radiographic hip OA was significantly less prevalent than that 10 years prior, especially among individuals aged between 40 and 69 years. How the prevalence of radiographic hip OA has decreased should be clarified, and the difference in the follow-up rates based on age may be attributable. In this study, the participants aged 40–49 years during the baseline survey had progressed to their 50s when the fourth survey was underway after 10 years. Interestingly, the participants aged 40–49 years during the baseline survey did not overlap with those aged 40–49 years during the fourth survey. Hence, we could analyze the trends in prevalence of hip OA over a 10-year period in each age-group as the birth cohort. Especially, the prevalence of radiographic hip OA for individuals in the age group of ≤60–69 years was different between the baseline and the fourth survey. In the fourth survey, in both men and women, 37.5%, 41.4%, 56.1%, and 83.7% of individuals aged 50–59, 60–69, 70–79, and 80–89 years, respectively, participated in the baseline survey. The percentage of new participants was high, especially those aged 50–69 years. In our previous study, the incidence of radiographic hip OA in 10 years was higher depending on the age [13], and the lower incidence of radiographic hip OA was associated with younger age of the individual. Therefore, we suggest that in patients aged ≤69 years, the prevalence of radiographic hip OA in a 10-year later survey was lower than that in the baseline survey. Whereas, the prevalence of radiographic hip OA in men aged 70–79 years in the fourth survey was significantly higher than that in men aged 70–79 years in the baseline survey. There are two possible reasons for this. First, 61.8% of the participants among men aged 70–79 years in the fourth survey participated in the baseline survey, and the percentage of the participants who already had radiographic hip OA in the baseline survey was high. Second, new participants might have a higher proportion of radiographic hip OA compared to younger age groups, because the incident hip OA had a hazard ratio of 1.05 (95% confidence interval, 1.03–1.08) at 1 year of age, which was significantly associated with age in our previous study [13].

**Fig. 3:**
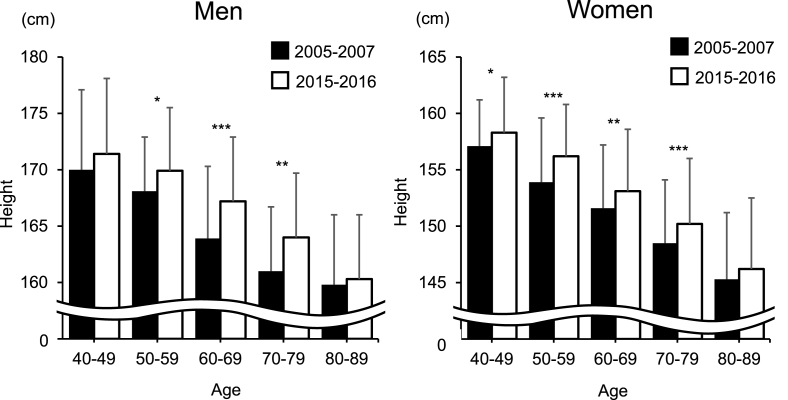
Height (cm) in men and women in each age strata (40–49, 50–59, 60–69, 70–79, and 80–89 years) at 2005–2007 and 2015–2016. ∗*p* ​< ​0.05, ∗∗*p* ​< ​0.01, ∗∗∗*p* ​< ​0.0001.

**Fig. 4:**
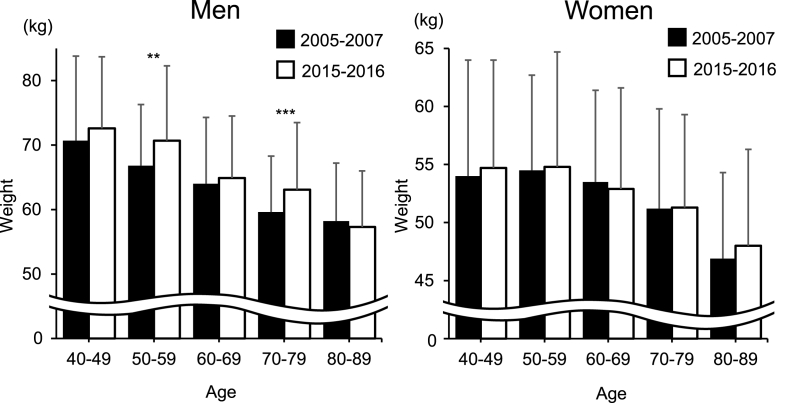
Weight (kg) in men and women in each age strata (40–49, 50–59, 60–69, 70–79, and 80–89 years) at 2005–2007 and 2015–2016. ∗∗*p* ​< ​0.01, ∗∗∗*p* ​< ​0.0001.

**Fig. 5:**
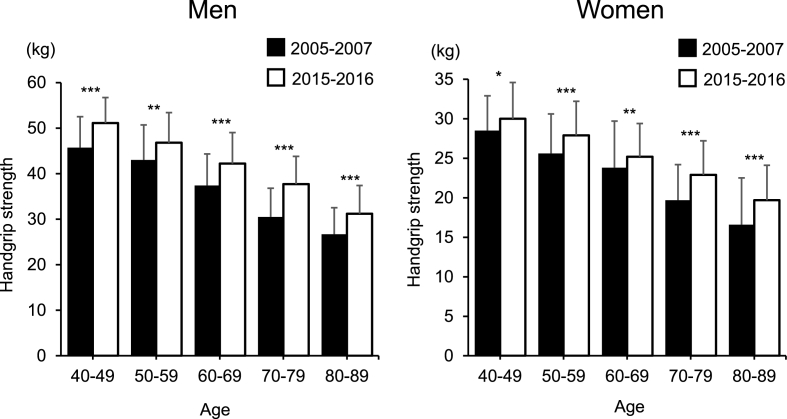
Maximum handgrip strength (kg) in men and women in each age strata (40–49, 50–59, 60–69, 70–79, and 80–89 years) at 2005–2007 and 2015–2016. ∗*p* ​< ​0.05, ∗∗*p* ​< ​0.01, ∗∗∗*p* ​< ​0.0001.

At this juncture, we questioned whether the decline in the prevalence of radiographic hip OA in individuals in the age group of ≤60–69 years is related to the trend in the anthropometric measurements and physical activities. In that regard, we noted that the height of Japanese individuals increased by ∼1 ​cm per decade for both sexes [24], which is primarily attributed to the influence of socioeconomic factors (ratio of workers in the primary, secondary, and tertiary industries to the total number of workers and gross national per capita expenditure at constant prices) and nutritional factors (per capita per day intake of nutrients and foods, energy–supply ratio from starchy foods, and protein supply ratio from animals) [24]. The National Health and Nutrition Survey (NHNS) was conducted in 2005 and 2015 by the Ministry of Health, Labor, and Welfare, Japan; in the 2005 survey, the mean heights of men aged 40–49, 50–59, 60–69, and 70–79 years were 169.6, 167.4, 164.2, and 161.0 ​cm, and those of women were 156.8, 154.6, 151.3, and 146.9 ​cm, respectively [25]. In the 2015 NHNS survey, the mean heights of men in the respective age groups were 170.8, 169.2, 167.0, and 162.0 ​cm, while those of women in the respective age groups were 158.0, 156.6, 153.3, and 148.5 ​cm [26]. Fig. 3 shows the age-sex distribution of height in the baseline and fourth surveys for this study; the mean height for each age group had a similar trend as that reported by the NHNS. However, Kouchi et al. revealed an increase in weight of men and a very low rate of weight increase in women [24]. From the 2005 NHNS study, the mean weight values of men in the respective age groups were 69.1, 66.6, 64.0, and 60.3 ​kg, while those of women were 55.1, 55.1, 53.9, and 49.7 ​kg, respectively [25]. In the 2015 NHNS, the mean weights of male participants were 70.6, 68.1, 66.3, and 61.0 ​kg, and those of women were 55.5, 55.0, 53.3, and 50.3 ​kg in the respective age groups [26]. Fig. 4 shows the age-sex distribution of weight in the baseline and the fourth surveys. The weight increase in each age stratum in the NHNS was slightly different from that in this study; however, the trend of the weight increase in 10 years in our study was consistent with that of NHNS. Moreover, the mean weights of women in 10 years were almost similar in both the NHNS and the present study. However, the tendency of the difference in height and weight in 10 years was inconsistent with that of the prevalence of radiographic hip OA in 10 years. Regarding the trends in handgrip strength, Fig. 5 shows the age-sex distribution in both the surveys. The handgrip strength of individuals aged 40–89 years was significantly higher in 2015–2016 than it was in 2005–2007 in our study. Suzuki et al. reported the mean handgrip strengths of men and women aged ≥65 years as 29.0–37.4 and 18.4–23.5 ​kg from measurements in 2007, respectively [15]. In the same study, the handgrip strengths measured in 2017 were 31.1–37.4 and 20.0–23.5 ​kg in men and women aged ≥65 years, respectively [15]. Moreover, Suzuki et al. reported that the handgrip strength of men aged ≥75 years and that of women aged ≥70 years had increased, which was similar to the trends of our study.

There are few reports concerning the association between hip OA and handgrip strength. The patients with sarcopenia with OA at the hip and lower limbs had significantly lower handgrip strength than those without OA [27]. Moreover, in patients with hip OA who underwent total hip arthroplasty, the pre-operative handgrip strength was positively associated with an increased improvement in function [28]. We investigated whether the decline in the prevalence of radiographic hip OA occurred because of the protective effect of increased muscle strength on the hip joint. Muscle dysfunction may be involved in the pathogenesis of OA [29]. Especially, Hootman et al. suggested that quadriceps weakness is an independent risk factor for hip or knee OA in a longitudinal study follow-up for 14.4 years [30]. Arokoski et al. reported that men with hip OA have significantly lower hip abduction, adduction, and flexion muscle strength than age- and sex-matched controls [31]. In our study, the trends in quadriceps strength over a 10-year period was not evaluated; however, the handgrip strength was found to be stronger than it was 10 years prior. Handgrip strength is strongly related to lower extremity muscle power and knee extension torque [32,33]. Ueoka et al. reported significantly less knee extension strength in the low handgrip strength group (≤18 ​kg) than in the normal handgrip strength group in patients awaiting total hip arthroplasty [34]. Therefore, in the present study, it is suggested that knee extension strength, especially quadriceps strength, increased more than the strength reported 10 years prior. Hence, stronger muscle strength may be a reducing factor for the prevalence of radiographic hip OA; however, additional consideration is needed to confirm this finding.

This study had a few limitations. It included independently living participants, instead of those living in institutional settings. Thus, the calculated prevalence of radiographic hip OA may have been underestimated. Although the ROAD study includes a large cohort, the participants in the present study may not be representative of the general population. In an earlier study, we compared the anthropometric measurements and lifestyle factors, such as smoking and drinking habits, between the study participants of the baseline survey of the ROAD study and the general Japanese population [21]. We found no significant differences between the two studies, except for the lower proportion of current smokers and drinkers in our study population than in the general Japanese population, suggesting that our study participants led healthier lifestyles. This selection survive bias should be considered when generalizing the results obtained from the present study. In addition, the number of male participants aged 40–49 years in the baseline survey and the fourth survey was small (44 and 60, respectively). The number of individuals with radiographic hip OA in men aged 40–49 years in the fourth survey was 0. Regarding selection bias, as we recruited new participants as volunteers and we included independently living participants, instead of those living in institutional settings, the “healthy” selection biases should be confirmed. However, the prevalence in men and women aged 40–49 and 50–59 years were considerably low. Therefore, we believe that the present study yields consistent results.

In conclusion, the trends in prevalence of hip OA over a 10-year period in two large-scale cross-sectional population-based cohort studies were reported. The prevalence of radiographic hip OA is showing an improving trend. This preferable change in circumstances could contribute to a decrease in the occurrence of radiographic hip OA in the future. Moreover, continued longitudinal surveys of the ROAD study will be useful to interpret the change in the prevalence of radiographic hip OA from 10 years prior for 3 years and the change of that 10 years later for 3 years, and will contribute to knowledge and potential prevention of hip OA.

## Author contributions

All authors have made substantial contributions to all three of the following:

(1) the conception and design of the study, acquisition of data, or analysis and interpretation of data.
(2) drafting the article or revising it critically for important intellectual content
(3) final approval of the version to be submitted

## Role of the funding source

This work was supported by JSPS KAKENHI Grant Number JP17H06628 and a Grant-in-Aid for the Japan Hip Joint Foundation (Director, Toshiko Iidaka). This study was also supported by a Grant–in-Aids for H17-Men-eki-009 (Director, Kozo Nakamura), H20-Choujyu-009 (Director, Noriko Yoshimura), H23-Choujyu-002 (Director, Toru Akune), H-25-Choujyu-007 (Director, Noriko Yoshimura), and H25-Nanchitou (Men)-005 (Director, Sakae Tanaka) of the 10.13039/501100003478Ministry of Health, Labor, and Welfare; and Scientific Research B23390172, B20390182, and Challenging Exploratory Research 24659317 to Noriko Yoshimura; B23390356, C20591774, and Challenging Exploratory Research 23659580 to Shigeyuki Muraki; Challenging Exploratory Research 24659666 and 21659349 and Young Scientists A18689031 to Hiroyuki Oka; B23390357 and C20591737 to Toru Akune; and Collaborating Research with NSF 08033011-00262 (Director, Noriko Yoshimura) from the Ministry of Education, Culture, Sports, Science, and Technology in Japan. This study was partly supported by grants from the Japan Agency for Medical Research and Development (17gk0210007h0003, Director, Sakae Tanaka), Japan Osteoporosis Society (Noriko Yoshimura, Shigeyuki Muraki, Hiroyuki Oka, and Toru Akune), and research aid from the Japanese Orthopedic Association (JOA-Subsidized Science Project Research 2006-1 & 2010-2; Director, Hiroshi Kawaguchi).

## Declaration of competing interest

There are no conflicts of interest.
